# Intranasal Administration of Disulfiram in Rats Produces Rapid and Potent Anxiolytic‐Like Effects Without Adverse Alcohol‐Related Interactions

**DOI:** 10.1155/adpp/5585693

**Published:** 2025-09-04

**Authors:** Arisa Ohta, Yuya Terashima, Kota Matsuura, Ryoji Fujizuka, Tasuku Hayashi, Kosei Nagai, Shintaro Shirakura, Tsugumi Yamauchi, Daisuke Yamada, Hiroshi Kinoshita, Kouji Matsushima, Akiyoshi Saitoh

**Affiliations:** ^1^ Laboratory of Pharmacology, Faculty of Pharmaceutical Sciences, Tokyo University of Science, Tokyo, 125-8585, Japan, tus.ac.jp; ^2^ Division of Molecular Regulation of Inflammatory and Immune Diseases, Research Institute for Biomedical Sciences (RIBS), Tokyo University of Science, Chiba, 278-0022, Japan, tus.ac.jp; ^3^ National Research Institute of Police Science, Kashiwa, Chiba, 277-0882, Japan, npa.go.jp

**Keywords:** alcohol deterrent drug, anxiolytic, depression, disulfiram, intranasal administration

## Abstract

**Background:** The U.S. Food and Drug Administration has approved disulfiram (DSF) as a treatment for alcoholism. DSF shows strong anxiolytic‐like effects in mice without the side effects commonly associated with benzodiazepines. However, when combined with alcohol, oral administration exhibits side effects, such as headache and decreased body temperature, limiting its clinical use. We aimed to evaluate the effects of intranasal DSF administration.

**Methods:** Male Wistar/ST rats were used. For intranasal formulation, DSF was encapsulated by 2‐hydroxypropyl‐β‐cyclodextrin to form an inclusion complex. The DSF formulation exhibited a uniform particle size. The elevated plus maze (EPM) test was used to examine the anxiety‐reducing effects of DSF. Ethanol (2000 mg/kg, p.o.) was administered 48 h after DSF. The body temperature was measured 2 h after ethanol administration. Subsequently, we collected blood and measured the blood acetaldehyde levels.

**Results:** Intranasal DSF administration (1.5 mg/rat) significantly increased the time spent in the open arms of the EPM within 20 min of administration. Oral DSF administration of DSF (1000 mg/kg) significantly increased the time spent in the open arms of the EPM 30 min after administration. In contrast to the oral administration, the intranasal DSF administration did not reduce body temperature or increase the blood acetaldehyde levels.

**Conclusions:** The intranasal DSF administration exhibited rapid and potent anxiolytic‐like effects at lower doses than oral administration, without triggering the alcohol interactions observed upon oral administration. Hence, the intranasal DSF formulation may have potential clinical applications as a novel anxiolytic agent.

**Trial Registration:** Japan Registry of Clinical Trials (jRCT): jRCTs031180183

## 1. Introduction

Disulfiram (DSF), a disulfide derivative of N, N‐diethyl dithiocarbonate, is a U.S. Food and Drug Administration–approved medication for treating alcoholism. Recently, we found that DSF effectively inhibits FROUNT [1], a cytoplasmic protein involved in C‐C chemokine receptor (CCR) 2 and CCR5 signaling pathways, which regulate directed migration and the infiltration of monocytes and macrophages [2–4]. In previous research, DSF was shown to block the interaction between CCR2/CCR5 and FROUNT, following a comprehensive screening of 131,200 compounds [1].

CCR2 and CCR5 signaling have been implicated in anxiety‐related behaviors. In a recent previous study, CCR2 knockout or antagonism has been shown to reduce stress‐induced anxiety and neuroinflammation [5, 6], and CCR5 inhibition mitigates anxiogenic responses caused by stress‐induced hippocampal CCR5/CCL5 expression [7]. Based on these findings, we now clearly state our hypothesis that DSF‐mediated inhibition of the CCR2/CCR5–FROUNT pathway may lead to anxiolytic‐like effects [8]. We unexpectedly observed an anxiolytic‐like effect of DSF while assessing secondary pharmacological effects [8]. This anxiolytic property was validated using the elevated plus maze (EPM) test, a standard method for screening anxiolytic drugs. DSF significantly increased the time spent in the open arms and the frequency of entries into them, without changing the overall number of entries. Unlike diazepam, DSF did not cause sedation in open‐field tests, disrupt coordination on the rotarod, or impair memory in the Y‐maze. These findings suggest that DSF may offer an effective, innovative anxiolytic option without the common side effects of benzodiazepines, such as memory impairment, coordination issues, and sedation, typically seen with diazepam. Moreover, DSF combined with an immune checkpoint inhibitor reduced tumor growth in mice via FROUNT inhibition, independent of its antialcoholism effects. A clinical trial is ongoing in gastric cancer patients.

However, the oral administration of DSF, especially with alcohol, is associated with adverse effects like headache and decreased body temperature due to its aldehyde dehydrogenase (ALDH) inhibition in the liver, limiting its clinical use [9, 10]. Oral administration is the most commonly used minimally invasive route of administration in clinical practice. Other delivery routes include transdermal, inhalation, and sublingual administration. Intranasal administration is a simple and minimally invasive route of administration, and as it is known as nose‐to‐brain, it is characterized by the ability to deliver compounds directly into the brain without first‐pass effects in the liver [11, 12]. Thus, we hypothesized that intranasal administration of DSF would reduce the side effects of oral administration.

Based on the aforementioned hypothesis, in this study, we aimed to examine the effects of intranasal administration on anxiety‐like behaviors in rats, along with the efficacy and adverse effects associated with oral and intranasal administration of DSF.

## 2. Materials and Methods

### 2.1. Animals

Behavioral experiments were conducted using male Wistar/ST rats (7 to 8 weeks old) obtained from Sankyo Labo Service Corporation, Inc. (Tokyo, Japan). A total of 159 rats were included. The animals were housed in an environment with a temperature of 23 ± 1°C and a 12‐h light/12‐h dark cycle. The lights were automatically turned on at 8:00 am. The rats were allowed *ad libitum* access to food and water. This research was conducted in compliance with the protocols approved by the Tokyo University of Science’s Institutional Animal Care and Use Committee (approval no. Y22014).

### 2.2. Drugs

DSF (Mitsubishi Tanabe Pharma Corporation, Osaka, Japan) was dissolved in 50% 2‐hydroxypropyl‐β‐cyclodextrin (HP‐β‐CD) (Wako Pure Chemical Industries, Ltd., Tokyo, Japan). DSF, or vehicle, was administered at a volume of 100 μL/rat. A Fine Particle Sprayer (FPS‐050‐A1, Toray Precision Co., Ltd., Shiga, Japan) was used for intranasal administration of the solution to the rats [13]. The drug was dissolved in 1% methylcellulose, which was also used as the vehicle. The drugs were administered orally to the rats via an animal‐specific disposable oral sonde (Fuchigami Instruments Ltd., Kyoto, Japan).

### 2.3. EPM Test

We conducted a modified version of the EPM test, as outlined in our previous study [14]. The EPM device comprises four arms arranged in a cross shape, extending from a central neutral area. The two opposing arms are enclosed by vertical walls, whereas the other two arms have open edges. The maze was positioned 70 cm above the floor under indirect lighting (35 lx). Prior to drug administration, the animals were acclimated in the experimental room for a minimum of 1 h. DSF (dose range: 0.1–1.5 mg/rat, i.n., *n* = 8–13) was administered 10, 20, 40, and 60 min before the EPM test. DSF (dose range: 250–1000 mg/rat, p.o., *n* = 5–8) was administered 15, 30, and 60 min before the EPM test.

Each rat was positioned in the neutral center zone facing a closed arm at the start of the 5‐min test session. A video camera system was used to observe and record the time spent in these areas. Data were analyzed using Smart 3.0 (Harvard Apparatus, Cambridge, MA, USA). An arm visit was recorded when the rat moved at least half of its body into the arm. The apparatus was cleaned after each animal test. The test measured the time spent in the open arms and total distance traveled.

### 2.4. Measurement of Body Temperature

The core temperature was measured by connecting the cable to a thermometer for rats (AD‐1687 Weighing Environment Logger, A and D). The timing of DSF and ethanol administration was determined by modifying previously described methods [9, 15]. Specifically, 40% ethanol (2000 mg/kg) was administered 48 h after DSF. 2 h later, the core temperature was measured. Three groups of rats were tested: control (no DSF, *n* = 5), oral (1000 mg/kg, *n* = 6), and intranasal (3.0 mg/rat, *n* = 6).

### 2.5. Determination of Blood Acetaldehyde Levels

We collected blood, and the blood acetaldehyde levels were measured 2 h after ethanol administration. Blood samples of 100 μL were promptly mixed with 500 μL of 0.6 N perchloric acid containing 0.001% (w/w) t‐butanol as an internal standard. The mixture was vortexed and centrifuged at 15,000 g for 3 min. Subsequently, 450 μL of the resulting supernatant was transferred to 20 mL gas chromatography (GC) vials for blood acetaldehyde level determination using GC. This study used a GC system with a flame ionization detector (GC‐2014, Shimazu, Kyoto, Japan) coupled with a headspace autosampler (TurboMatrix 40, PerkinElmer, Waltham, MA, USA). The chromatographic parameters were as follows: Column temperature was set at 90°C, injector at 110°C, and detector at 200°C. A Supelcowax wide bore capillary column (60 m length, 0.53 mm i.d., 2 μm film thickness, Supelco, PA, USA) was used for separation. Nitrogen served as the carrier gas at a pressure of 50 kPa [16].

### 2.6. Particle Size Distribution (PSD)

Microtrac AEROTRAC II (Microtrac Inc., Montgomeryville, PA, USA) was used to determine the PSD of the samples [17]. The equipment provides PSD in the range of 0.020–2000 μm.

### 2.7. Data Analysis

The results are presented as mean values with standard errors of the mean (SEM). A one‐way analysis of variance (ANOVA) was used for comparisons between more than two groups; a one‐way ANOVA was employed. Subsequent pairwise comparisons between individual groups were performed using Bonferroni’s multiple comparison test. All statistical analyses were performed using the GraphPad Prism software (GraphPad Software Inc., San Diego, CA, USA). Statistical significance was set at *p* < 0.05.

All behavioral scoring and data analysis were performed by an experimenter who was blinded to the treatment allocation until the completion of data analysis.

## 3. Results

### 3.1. Characterization of DSF for Intranasal Formulation

**Figure 1:**
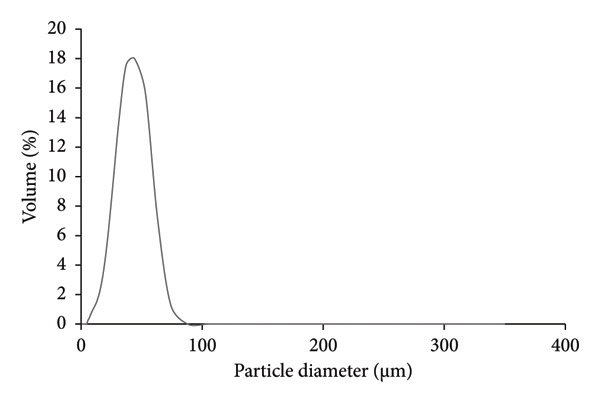
Characterization of DSF. The diameters of the particles in the DSF spray (15 mg/mL). (*n* = 3).

For intranasal formulation, DSF was encapsulated by HP‐β‐CD to form an inclusion complex. DSF at 15 mg/mL in 50% HP‐β‐CD produced particles with a mean size of 28.29 ± 13.35 μm (Figure 1), which were homogeneously dispersed and considered suitable as nanoparticles for intranasal administration.

### 3.2. EPM Test

#### 3.2.1. Intranasal Administration

Figure 2(a) shows representative examples of the locomotor paths in the EPM test (open arms in the vertical direction). At 20 min after administration, DSF exhibited a significant dose‐dependent effect, increasing the duration that the rats remained in the open arms (*F* [3, 29] = 3.441, *p* = 0.0296). Bonferroni analysis revealed a significant effect of DSF compared to that in the vehicle‐treated group (0.1 mg/rat, i.n.: *t* = 0.01978, *p > *0.05; 0.5 mg/rat, i.n.: *t* = 1.407, *p > *0.05; 1.5 mg/rat, i.n.: *t* = 2.7, *p* = 0.0343; Figure 2(b)). DSF at any dose (0.1–1.5 mg/rat) had no significant effect on total distance (*F* [3, 29] = 0.8403, *p* = 0.4829) (Figure 2(c)). Thus, it seems that no sedating effects were observed at the administered doses.

Figure 2Anxiolytic‐like effects of DSF administered intranasally to rats as observed in the elevated plus maze test. (a) Representative activity traces in the EPM test of vehicle‐treated and DSF‐treated rats. (b) The percentage of time spent in the open arms. (c) Total distance. DSF and the vehicle were administered 20 min before the test. The columns represent the following in order: vehicle (*n* = 8), DSF 0.1 mg/rat (*n* = 8), DSF 0.5 mg/rat (*n* = 8), and 1.5 mg/rat (*n* = 9). Each column represents the mean ± SEM. The statistical significance of differences among the DSF treatment groups was assessed using one‐way ANOVA. Post hoc individual group comparisons were made with Bonferroni’s test. Statistical significance is denoted by ^∗^ *p* < 0.05, respectively, versus vehicle‐treated rats.

**Figure:**
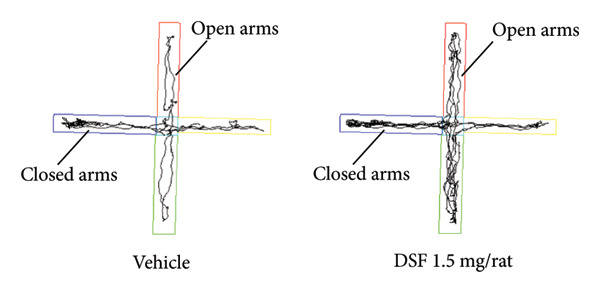
(a)

**Figure:**
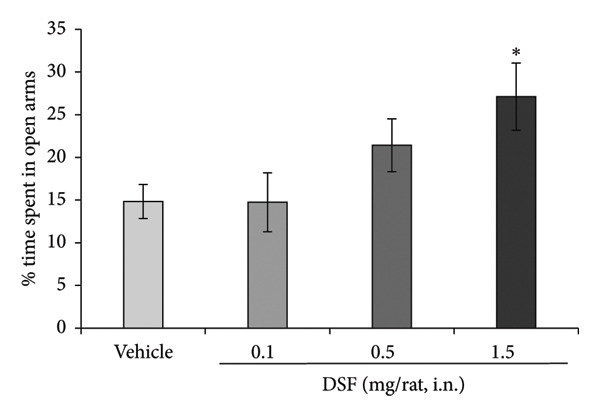
(b)

**Figure:**
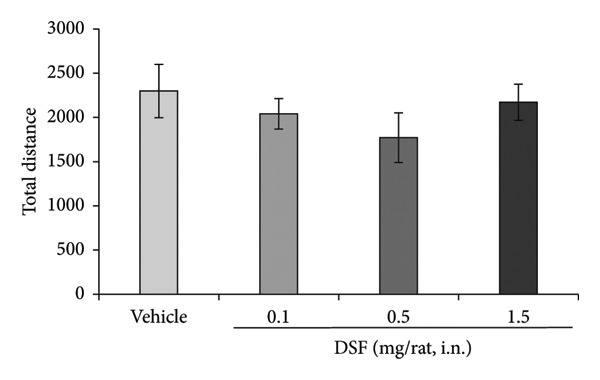
(c)

Figure 3(a) demonstrated that DSF (1.5 mg/rat) significantly increased the percentage of time spent in the open arms at 10 and 20 min postadministration (*F* [4, 52] = 2.613, *p* = 0.0458). The peak effect on open‐arm duration was observed at 20 min following DSF administration (10 min: *t* = 2.721, *p* = 0.0354; 20 min: *t* = 2.814, *p* = 0.0276; 40 min: *t* = 2.051, *p* > 0.05; 60 min: *t* = 1.694, *p* > 0.05; Figure 3(a)). However, DSF did not significantly impact the total distance traveled at any time point from 10 to 60 min (*F* [4, 52] = 1.186, *p* = 0.3280) (Figure 3(b)), suggesting no apparent sedative effects at these dosages.

Figure 3Anxiolytic‐like effects of DSF administered intranasally to rats as observed in the elevated plus maze test. (a) The percentage of time spent in the open arms and (b) total distance covered following DSF (1.5 mg/rat, i.n.) administration. DSF was administered 10, 20, 40, and 60 min before the test. The columns represent the following in order: vehicle (*n* = 12), DSF 10 min (*n* = 13), DSF 20 min (*n* = 10), DSF 40 min (*n* = 11), and DSF 60 min (*n* = 11). Each column represents the mean ± SEM. The statistical significance of differences among the DSF treatment groups was assessed using one‐way ANOVA. Post hoc individual group comparisons were made with Bonferroni’s test. Statistical significance is denoted by ^∗^ *p* < 0.05, respectively, versus vehicle‐treated rats.

**Figure:**
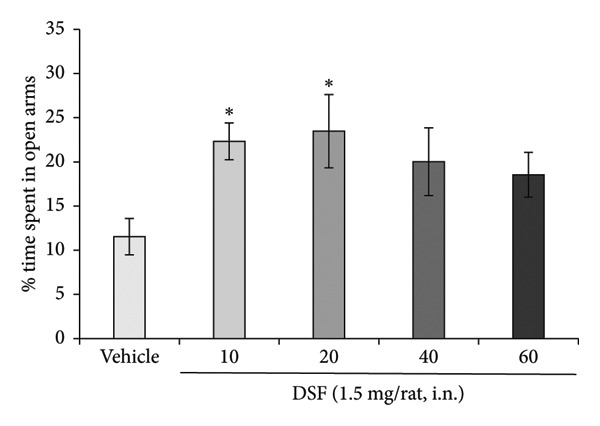
(a)

**Figure:**
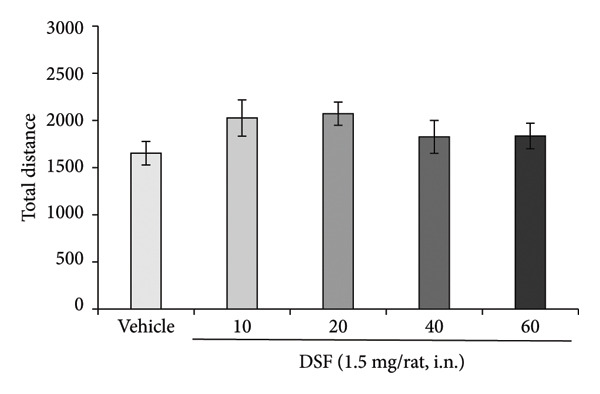
(b)

#### 3.2.2. Oral Administration

DSF significantly and dose‐dependently increased the time spent by rats in the open arms 30 min after administration (*F* [3, 18] = 4.382, *p* = 0.0175), as revealed by Bonferroni analysis compared to the vehicle‐treated group (250 mg/kg, p.o.: *t* = 2.452, *p* > 0.05; 500 mg/kg, p.o.: *t* = 2.170, *p* > 0.05; 1000 mg/kg, p.o.: *t* = 3.480, *p* = 0.008; Figure 4(a)). DSF at any dose (250–1000 mg/kg) had no significant effect on total distance traveled (*F* [3, 18] = 1.978, *p* = 0.1534) (Figure 4(b)), suggesting no apparent sedative effects at the dosages used.

Figure 4Anxiolytic‐like effects of DSF delivered p.o. to rats as observed in the elevated plus maze test. (a) The percentage of time spent in the open arms. (b) Total distance covered. DSF and the vehicle were administered 30 min before the test. The columns represent the following in order: vehicle (*n* = 6), DSF 250 mg/kg (*n* = 5), 500 mg/kg (*n* = 6), and 1000 mg/kg (*n* = 5). Each column represents the mean ± SEM. The statistical significance of differences among the DSF treatment groups was assessed using one‐way ANOVA. Post hoc individual group comparisons were made with Bonferroni’s test. Statistical significance is denoted by ^∗^ *p* < 0.05 and ^∗∗^ *p* < 0.01, respectively, versus vehicle‐treated rats.

**Figure:**
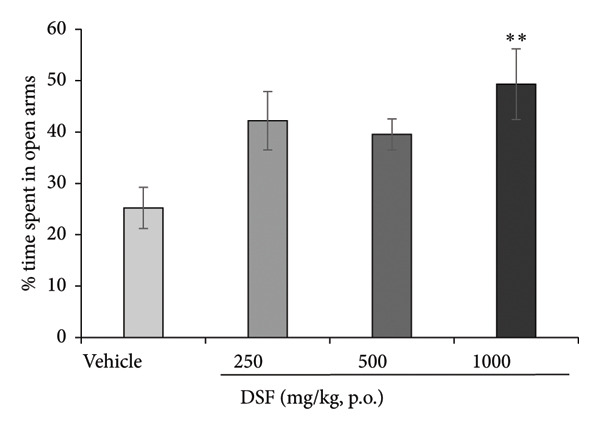
(a)

**Figure:**
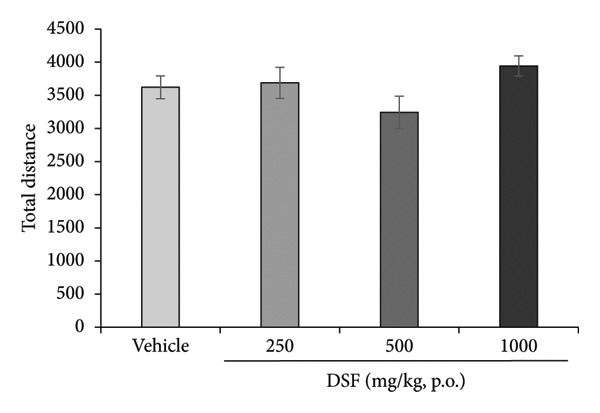
(b)

As shown in Figure 5(a), DSF (1000 mg/kg) significantly increased the percentage of time spent in the open arms 30 min after administration (*F* [3, 26] = 3.544, *p* = 0.0283). The peak effect on time spent in the open arms was noted 30 min after DSF administration (10 min: *t* = 0.1686, *p* > 0.05; 30 min: *t* = 2.807, *p* = 0.028; 60 min: *t* = 1.791, *p > *0.05; Figure 5(a)). DSF at any time (10–60 min) had no significant effect on the total distance traveled (*F* [3, 26] = 0.1246, *p* = 0.9447) (Figure 5(b)), suggesting no apparent sedative effects at the dosages used.

Figure 5Anxiolytic‐like effects of DSF delivered p.o. to rats as observed in the elevated plus maze test. (a) The percentage of time spent in the open arms and (b) total distance covered following DSF (1000 mg/rat, p.o.) administration. DSF was administered 10, 30, and 60 min before the test. The columns represent the following in order: vehicle (*n* = 8), DSF 10 min (*n* = 7), DSF 30 min (*n* = 8), and DSF 60 min (*n* = 7). Each column represents the mean ± SEM. The statistical significance of differences among the DSF treatment groups was assessed using one‐way ANOVA. Post hoc individual group comparisons were made with Bonferroni’s test. Statistical significance is denoted by ^∗^ *p* < 0.05 respectively, versus vehicle‐treated rats.

**Figure:**
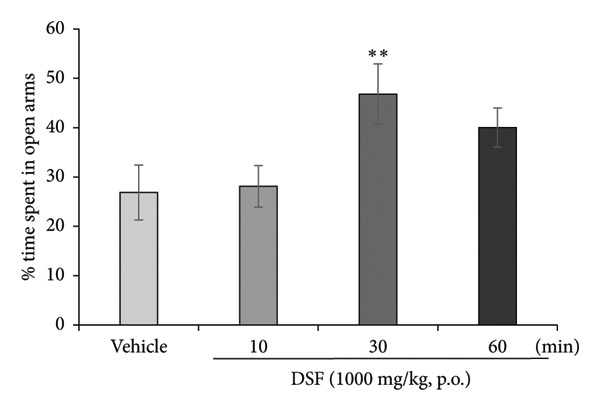
(a)

**Figure:**
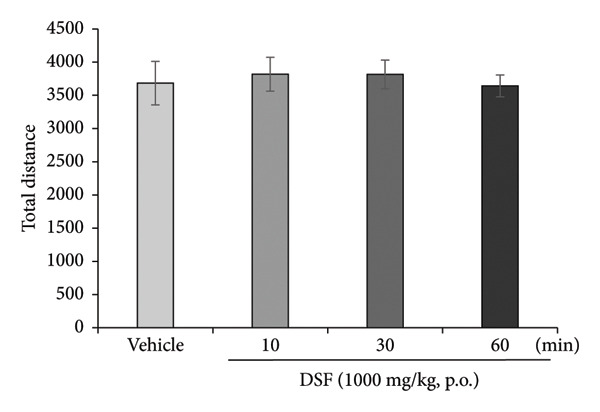
(b)

### 3.3. Side Effects

Figure 6(a) shows the time course of administration. A significant decrease in body temperature was observed in the oral DSF group compared to that in the control Group 2 h after ethanol administration. (*F* [2, 14] = 6.25, *p* = 0.0115). However, no differences were noted in body temperature in the intranasal DSF group. Bonferroni analysis revealed a significant decrease in body temperature of oral DSF in the treatment groups when compared to the control group (1000 mg/kg, p.o.: *t* = 2.632, *p* = 0.0394, 3.0 mg/rat, i.n. *t* = 0.5423, *p* > 0.05; Figure 6(b)).

Figure 6Side effects of DSF. (a) Timeline of drug administration. Rats were administered DSF (1000 mg/kg, p.o.; 3.0 mg/rat, i.n.) and ethanol (2000 mg/kg, p.o.) after 48 h. Control rats were administered only ethanol. (b) The core temperature was measured 2 h after ethanol administration. (c) Blood samples were obtained 2 h after the ethanol treatment, and acetaldehyde concentrations were determined. The columns represent the following in order: control (*n* = 5), DSF 1000 mg/kg, p.o. (*n* = 6), DSF 3.0 mg/rat, and i.n. (*n* = 6). Each column represents the mean ± SEM. The statistical significance of differences among the DSF treatment groups was assessed using one‐way ANOVA. Post hoc individual group comparisons were made with Bonferroni’s test. Statistical significance is denoted by ^∗^ *p* < 0.05 and ^∗∗^ *p* < 0.01, respectively, vs. control rats.

**Figure:**
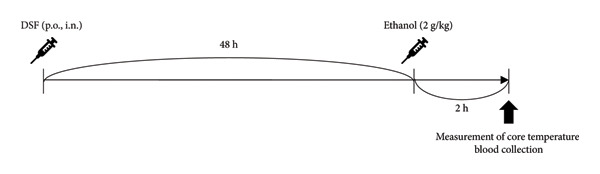
(a)

**Figure:**
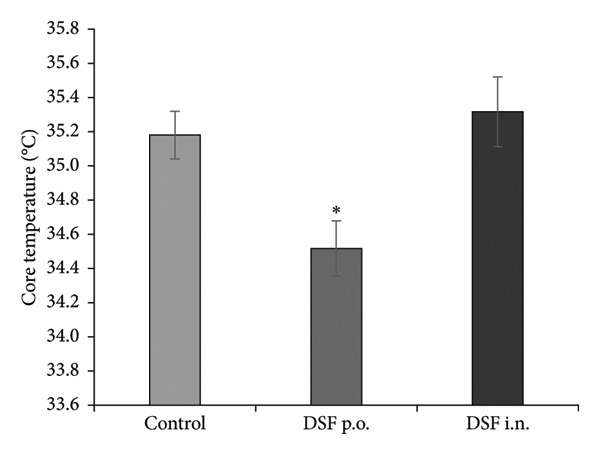
(b)

**Figure:**
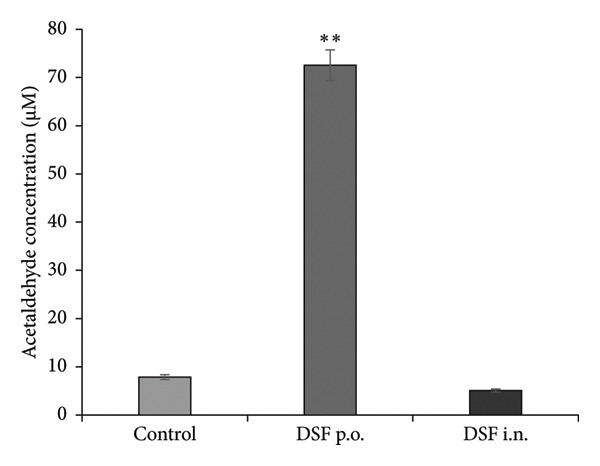
(c)

In addition, a significant increase in blood acetaldehyde concentration was observed in the oral DSF group compared to that in the control group (*F* [2, 14] = 379.3, *p* < 0.0001). However, no differences were noted in blood acetaldehyde concentrations in the intranasal DSF group. Bonferroni analysis revealed an increase in blood acetaldehyde concentration of DSF in the treatment groups when compared to the control group (1000 mg/kg, p.o.: *t* = 22.55, *p* < 0.0001, 3.0 mg/rat, i.n. *t* = 0.962, *p* > 0.05; Figure 6(c)).

## 4. Discussion

In the present study, DSF was used to develop an intranasal formulation with a uniform particle size. The intranasal formulation showed rapid anxiolytic‐like effects in rats, and these effects were more potent than those observed with oral administration. In contrast, the intranasally administered DSF demonstrated significantly fewer side effects than those of orally administered DSF.

### 4.1. Physical Properties of HP‐β‐CD‐Conditioned Preparations

The PSD of DSF in the spray state was uniform, and its mean particle size was 28.29 ± 13.35 μm (Figure 2(a)). Particle size affects drug deposition in the nasal mucosa. For liquid metered‐dose atomizers, a particle size of 30–60 μm is optimal. Therefore, the particle size of DSF/HP‐β‐CD was suggested to be approximately appropriate for intranasal formulation. Furthermore, although DSF was almost insoluble in water, we succeeded in improving its insolubility by dissolving it in a 50% HP‐β‐CD solution. The DSF/HP‐β‐CD inclusion complex was prepared to significantly enhance the poor aqueous solubility of DSF, achieving an approximate 2450‐fold improvement [18]. We hypothesize that the physical stability of DSF dissolved in HP‐β‐CD solution results in a more uniform particle size of the intranasal formulation.

### 4.2. Anxiolytic‐Like Effects Observed With Intranasal Administration

Intranasal administration of DSF in rats significantly increased the time spent in the open arm in a dose‐dependent manner, suggesting anxiolytic‐like effects. A dose of 6.3 ± 0.092 mg/kg per body weight of DSF showed significant effects with intranasal administration (1.5 mg/rat). Male rats with glioma were treated with DSF via intranasal administration (10 mg/kg, i.n.) for 5 days [18, 19], indicating that the therapeutic potential of DSF against C6 glioma in intracranial glioma‐bearing male rats was achieved at 10 mg/kg with intranasal administration. The administration concentration of DSF that showed an anxiolytic‐like effect in this study was approximately the same as that previously reported, which was effective for intracranial glioma‐bearing male rats, suggesting that the concentration at which the effect was observed in this experiment is reasonable.

Additionally, the previously mentioned study proposed that the tumor tissues in model rats treated via nasal drug administration were the least aggressive, indicating that the DSF/HP‐β‐CD/Cu intranasal formulation effectively inhibited tumor migration when delivered via the nose‐to‐brain route. The present study showed that oral administration of DSF to rats increased the time spent in the open arm in a dose‐dependent manner, suggesting anxiolytic‐like effects at 1000 mg/kg. As noted above, intranasal administration of DSF elicited anxiolytic‐like effects at a dose of 1.5 mg/rat. Indeed, EPM tests showed that rats administered intranasal DSF exhibited anxiolytic‐like effects at doses 160 times lower than those administered orally. This could be attributed to the following reasons: DSF is metabolized in the stomach when administered orally [20]. It is also expected to improve bioavailability by avoiding drug degradation and metabolism in the stomach and intestines and avoiding hepatic first‐pass effects. A previous study indicated that intranasal quercetin nanogels exhibited a 50‐fold enhanced higher bioavailability compared to oral quercetin since intranasal administration is associated with improved bioavailability compared to oral administration [21]. In addition, intranasal administration of DSF showed a potent anxiolytic‐like effect within 10 and 20 min after administration, whereas oral administration of DSF required 30 min to induce significant anxiolytic effects, suggesting that intranasal administration of DSF results in more immediate induction of anxiolytic‐like effects than oral administration. The nasal route offers a noninvasive method for administering pharmaceutical compounds that target local and central nervous system (CNS) effects. Drugs absorbed directly through the trigeminal and olfactory pathways in the nasal cavity gain immediate access to the brain, resulting in improved pharmacokinetic/pharmacodynamic (PK/PD) profiles for CNS‐acting drugs [22]. This administration route presents a promising alternative to enteral and systemic methods for delivering potent and effective CNS‐targeted drugs to the brain parenchyma, circumventing major physiological barriers, such as the blood–brain barrier (BBB) and blood–cerebrospinal fluid barrier (BCSFB). Compared to oral or intravenous administration, nasal drug delivery offers several benefits, including noninvasiveness, ease of self‐administration, quicker onset of action, and enhanced bioavailability by avoiding hepatic first‐pass metabolism. Additionally, bypassing the BBB may potentially increase drug availability in the CNS [22, 23]. Considering these factors, intranasal administration has been proposed as a rapid delivery method because the BBB poses the primary obstacle for systemically administered therapeutics to reach the CNS.

As shown in Figure 3, the anxiolytic‐like effect of intranasal DSF peaks approximately 20 min after administration and then diminishes over the subsequent 40–60 min. This pattern suggests that DSF may act rapidly in the CNS, potentially via direct nose‐to‐brain transport that bypasses first‐pass metabolism. However, the transient nature of the effect raises important considerations for clinical application. Specifically, the short duration of action may limit its therapeutic utility unless the dosing regimen is optimized. Repeated administration or the development of a sustained‐release intranasal formulation could help maintain effective drug levels and prolong the anxiolytic effect. Future studies should investigate pharmacokinetic profiles alongside behavioral outcomes to assess the feasibility and safety of these strategies.

We previously reported that the intraperitoneal administration of 40 mg/kg of DSF exerts anxiolytic‐like effects in mice [8]. However, in the present study, oral administration of 1000 mg/kg of DSF was effective in rats. The first reason for the difference in dosage is the species‐specific differences between mice and rats. Another reason for this discrepancy is the difference in the route of administration. DSF is believed to be metabolized in the liver and stomach to exert its original effect of ALDH inhibition. Therefore, in this study, the oral formulation used was hypothesized to be metabolized in the stomach and did not exhibit anxiolytic‐like effects at low doses. ALDH inhibition, a therapeutic mechanism influencing alcohol dependence, has been demonstrated in rats following oral administration of 1000 mg/kg of DSF [15]. Therefore, in the present study, anxiolytic‐like effects were observed at the same dose.

### 4.3. Evaluation of Adverse Effects After Intranasal Administration

The side effects observed when DSF was administered orally were not observed with intranasal administration. In this study, anxiolytic‐like effects were evaluated 30 min after oral administration of DSF, whereas side effects were evaluated 50 h after DSF administration. The timing difference was employed because the inhibitory effects on FROUNT and ALDH inhibitory are noted at different times. Intraperitoneal administration of DSF in rodent models has demonstrated FROUNT inhibitory effects within 15–60 min [8, 15], whereas ALDH inhibitory effects are noted after 8–50 h [24]. Therefore, in this study, we performed the test at 50 h after DSF administration, in consistency with previous studies.

Intranasal administration of DSF did not increase blood aldehyde concentrations or decrease body temperature. These results suggested that the ALDH inhibitory effect of DSF was not observed after intranasal administration. The inhibitory effect of ALDH is strongly influenced by DSF metabolites in the liver [24, 25]. Intranasal administration was performed at a dose of 3 mg/rat, twice the dose of 1.5 mg/rat that showed anxiolytic‐like effects. However, no adverse effects were observed. These results suggest that the intranasal administration of DSF is a safer formulation for reducing systemic side effects.

Although the pathways that transport drugs from the nasal cavity to the brain have not yet been fully elucidated, three major pathways are hypothesized to be involved. The first pathway involves transport both between cells and between cells and nerve bundles/neurons. In the second route, the drug is absorbed into the bloodstream and transported throughout the body to the brain. The third pathway involves uptake within the olfactory and trigeminal nerves and transport to the brain [26]. Cy5.5/HP‐β‐CD exhibited strong brain targeting via nose‐to‐brain transport, demonstrated by significantly higher fluorescence intensity in the brain compared to other organs [18]. Further investigation of the relation between particle deposition in the nasal cavity and analysis of the route of administration are necessary. We suggest that nose‐to‐brain delivery provides enhanced therapeutic benefits even in the low doses. This method offers several advantages including escaping liver metabolism, enabling targeted drug delivery, providing a faster onset of action, increasing the surface area for drug absorption, and reducing systemic exposure.

### 4.4. Clinical Application Potential With DSF Intranasal Formulation

As described above, intranasal administration has emerged as a superior route of administration to effectively deliver test drugs to the CNS; however, several challenges are required to be addressed. The development of intranasal formulations for the treatment of CNS diseases, such as Alzheimer’s disease, Parkinson’s disease, and brain tumors, has been investigated because of the potential for increased brain transfer by changing to an intranasal formulation [27]. We have previously shown that intranasal administration of a derivative of oxytocin, a neuropeptide, in mice delivers the oxytocin derivative to limbic regions, such as the hypothalamus and hippocampus, 20 min after administration, and the pharmacological effects of intranasal administration are comparable to those of intracerebroventricular administration [28].

## 5. Conclusion

We hypothesized that intranasal administration of DSF offers several benefits over oral delivery, including superior central reach and pharmacological effects, as well as reduced peripheral side effects. In this study, we observed that intranasal DSF may indeed have advantages over oral administration. Future research should focus on PK/PD studies to clarify DSF levels and drug effectiveness across different brain regions following intranasal delivery.

Based on the present results, we demonstrated that intranasal DSF is an effective and safe route of administration that does not exhibit the alcohol interactions observed with the oral administration of DSF. Our research demonstrates that intranasal administration of DSF is an effective and promising formulation for clinical development as a psychotropic drug. In the future, it will be desirable to evaluate the safety of DSF formulations.

**Nomenclature**
ANOVA: Analysis of variance
ALDH: Aldehyde dehydrogenase
BBB: Blood–brain barrier
CNS: Central nervous system
DSF: Disulfiram
EPM: Elevated plus maze
GC: Gas chromatography
HP‐β‐CD: Hydroxypropyl‐β‐cyclodextrin
PK/PD: Pharmacokinetic/pharmacodynamic
PSD: Particle size distribution

## Conflicts of Interest

The authors declare no conflicts of interest.

## Author Contributions

The conceptualization of the study was carried out by Akiyoshi Saitoh, Yuya Terashima, and Kouji Matsushima. The methodology was developed by Kota Matsuura, Ryoji Fujizuka, Tasuku Hayashi, Kosei Nagai, Shintaro Shirakura, and Tsugumi Yamauchi. Formal analysis and investigation were conducted by Arisa Ohta. The original draft preparation was done by Arisa Ohta and Akiyoshi Saitoh, while the review and editing of the manuscript were handled by Akiyoshi Saitoh and Daisuke Yamada. Funding acquisition was secured by Arisa Ohta. Resources for the study were provided by Hiroshi Kinoshita, and supervision was managed by Akiyoshi Saitoh.

## Funding

This work was partially supported by a Grant‐in‐Aid for JSPS Fellows (Grant no.JP24KJ2020 to Arisa Ohta).

## Data Availability

The data that support the findings of this study are available from the corresponding author upon reasonable request.
